# Efficient Bioelectrochemical Conversion of Industrial Wastewater by Specific Strain Isolation and Community Adaptation

**DOI:** 10.3389/fbioe.2019.00023

**Published:** 2019-02-19

**Authors:** Stefanie Brunner, Tina Klessing, Andreas Dötsch, Katrin Sturm-Richter, Johannes Gescher

**Affiliations:** ^1^Department of Applied Biology, Institute for Applied Biosciences, Karlsruhe Institute of Technology, Karlsruhe, Germany; ^2^Department of Physiology and Biochemistry of Nutrition, Max Rubner-Institut, Federal Research Institute of Nutrition and Food, Karlsruhe, Germany; ^3^Department of Biofilm Technologies, Institute for Biological Interfaces 1, Karlsruhe Institute of Technology, Eggenstein-Leopoldshafen, Germany

**Keywords:** bioelectrochemical systems (BES), wastewater, *Geobacter sulfurreducens*, microbial community, coculture, TOC removal

## Abstract

The aim of this study was the development of a specifically adapted microbial community for the removal of organic carbon from an industrial wastewater using a bioelectrochemical system. In a first step, ferric iron reducing microorganisms were isolated from the examined industrial wastewater. In a second step, it was tested to what extent these isolates or a cocultivation of the isolates with the exoelectrogenic model organism *Geobacter sulfurreducens* (*G. sulfurreducens*) were able to eliminate organic carbon from the wastewater. To establish a stable biofilm on the anode and to analyze the performance of the system, the experiments were conducted first under batch-mode conditions for 21 days. Since the removal of organic carbon was relatively low in the batch system, a similar experiment was conducted under continuous-mode conditions for 65 days, including a slow transition from synthetic medium to industrial wastewater as carbon and electron source and variations in the flow rate of the medium. The overall performance of the system was strongly increased in the continuous- compared to the batch-mode reactor and the highest average current density (1,368 mA/m^2^) and Coulombic efficiency (54.9%) was measured in the continuous-mode reactor inoculated with the coculture consisting of the new isolates and *G. sulfurreducens*. The equivalently inoculated batch-mode system produced only 82-fold lower current densities, which were accompanied by 42-fold lower Coulombic efficiencies.

## Introduction

Over the past decades, the demand of freshwater increased considerably. Social and economic growth and the increase in world population result not only in an increasing freshwater demand but also in a rising energy need (UN-WWAP, 2015; IEA, 2017; Roser and Ortiz-Ospina, 2017). Microbial fuel cells are bioelectrochemical systems (BES) that generate electrical energy and since waste streams like wastewater accrue in large amounts, these systems gained much attention in the recent years because they offer a technological possibility to couple the elimination of organic carbon to the production of electrical energy (Winter and Brodd, 2004).

Wastewater contains a high amount of energy in the form of organic matter, which is not used in conventional water treatment plants. In contrary, elimination of this organic carbon is the most energy demanding step in wastewater treatment as it usually necessitates the supply of oxygen as electron acceptor (Heidrich et al., 2011; Gude, 2016). The application of microbial fuel cell (MFC) technology for wastewater treatment would overcome this problem because anodes instead of oxygen are used here as terminal electron acceptor of microbial respiration. The released respiratory electrons are transferred via an external circuit to the cathode where oxygen is reduced.

Anodic respiration necessitates the development of an extended respiratory chain to the cell surface (Lovley, 2006; Amos et al., 2007; Schröder, 2007; TerAvest and Ajo-Franklin, 2016). *Shewanella* and *Geobacter* species are the best studied anode-reducing (exoelectrogenic) microorganisms (Logan, 2009; Kumar et al., 2016). Multiheme cytochromes play key roles as catalysts for extracellular electron transfer, as they catalyze the electron transfer in multiple steps from the cytoplasmic membrane to the cell surface (Mehta et al., 2005; Schröder, 2007). The terminal reductases are not specific and therefore the organisms can reduce many different materials including graphite electrodes as long as they are poised to a suitable redox potential (Beliaev et al., 2005; Methé et al., 2005; Logan, 2009). Besides organisms belonging to the genera *Geobacter* and *Shewanella*, many other bacteria have been identified as exoelectrogenic organisms, as for instance different Clostridium strains (Park et al., 2001; Zhang et al., 2012; Kumar et al., 2016). However, it is often unclear how these organisms conduct extracellular electron transfer.

Using MFC-technology and exoelectrogenic bacteria for the treatment of industrial wastewater is of particular interest because of the stable composition and generally higher concentration of organic compounds in industrial wastewater (Agler-Rosenbaum et al., 2013; Gude, 2016). Both factors would theoretically allow for stable process conditions and high current densities. Literature provides different examples of industrial wastewaters that have been analyzed in MFCs regarding current production and carbon degradation. The efficiency of the MFCs differs drastically depending on the type of wastewater, the operation conditions and the reactor setup (Logan et al., 2006; Gude, 2016). For instance, while the treatment of dairy wastewater resulted in a current density of 0.79 A/m^2^ and a COD removal of more than 90% (Mansoorian et al., 2016), a MFC fed with paper wastewater produced only 125 mA/m^2^ and only 78% of the organic carbon was consumed (Velasquez-Orta et al., 2011). Hence, the question arises whether or how bioelectrochemical systems could be adapted to the specific needs that result from the individual wastewater stream. This study follows the idea of a biological adaptation of the anode community as an instrument to increase process efficiency.

Here, industrial wastewater of a chemical park was used as carbon and electron source for exoelectrogenic microorganisms in a BES. The ability of microorganisms to reduce ferric iron is very often (but not always) an indication that these organisms can also produce current in a BES (Richter et al., 2012). Therefore, ferric iron reducing microorganisms were isolated in a first step from the industrial wastewater and their ability to reduce ferric was quantified. Together with the lab strain *Geobacter sulfurreducens* PCA, their performance was analyzed in a bioelectrochemical reactor regarding current production, Coulombic efficiency (CE) and total organic carbon (TOC) elimination. Since the initial activity of the microorganisms was rather low, we established an adaptation routine that lead to roughly 90-fold increased current production rates. A metatranscriptomic study was conducted to understand the adaptation of the organisms on a molecular level and to analyze the interaction within the microbiome.

## Materials and Methods

### Isolation and Cultivation of Exoelectrogenic Bacteria

The organisms characterized in this study were isolated from the wastewater of a chemical park. The isolation was carried out by serial dilutions and spread plate technique using a synthetic ferric citrate-medium designed for growth of exoelectrogenic microorganisms modified from Dolch et al. (2014). Electron acceptor and donors were used in the following concentrations: 10 mM sodium acetate, 20 mM lactate and 4.4 mM sodium propionate as electron donors and 40 mM Fe(III)-citrate as electron acceptor. The medium was flushed with N_2_/CO_2_ (80%/20%) for 30 min to remove the dissolved oxygen. For spread plate technique, the medium was supplemented with 2% agar. The incubation temperature was 37°C.

### Anaerobic Growth of the Strains and Fe(III)-Reduction

For growth experiments, the isolates were incubated at 37°C and *G. sulfurreducens*_bc_ (laboratory barcode strain, described by Dolch et al. (2015) at 30°C. The isolated *Escherichia coli* strain was grown in ferric citrate medium as described above. The isolated organisms that were most closely related to *Clostridium sartagoforme, Clostridium butyricum*, and *Paenibacillus phoenicis* were cultivated in ferric citrate medium with 50 mM glucose as electron donor and carbon source. *G. sulfurreducens*_bc_ was cultivated in ferric citrate medium with 40 mM disodium fumarate as electron acceptor. The initial OD_655_ was 0.05. For adaptation experiments, *G. sulfurreducens*_bc_ was grown in medium composed of different ratios of wastewater and synthetic medium. Electron acceptor and donor concentrations in the adaptation media were adjusted to 21 mM acetate and 40 mM fumarate, respectively.

### Wastewater

The wastewater was stored at −20°C. Prior to use, it was sterile filtered using a pore size of 0.2 μm. Oxygen was removed from the wastewater by repeatedly flushing the headspace of the bottle for 2 min with N_2_, followed by a vacuum application for another 2 min. Nitrogen gas and vacuum cycles were repeated for 30 min.

### BES Experiments

#### Bioelectrochemical Three-Electrode Setup

BES were operated in a two-chamber setup with a volume of 270 mL (anodic compartment) and an anode size of 36 cm^2^ as described in Förster et al. (2017). A graphite felt (GFD 2.5 EA; SGL Group, Carbon Company; Germany) served as working electrode material and a platinum mesh with a size of 0.5 cm^2^ was used as counter electrode. Before use, the anode was rinsed with isopropanol first, followed by deionized water. The entire bioelectrochemical setup was sterilized by autoclaving.

The anode potential was adjusted to 0 mV vs. normal hydrogen electrode (NHE) with a potentiostat (6 EmStat3, PalmSens BV; Netherlands) and Ag/AgCl reference electrodes (-199 mV vs. Ag/AgCl) (Sensortechnik Meinsberg GmbH; Germany). The medium in the reactor was stirred at 120 rpm using a magnetic stir bar.

#### Strain Treatment and Cultivation Routine

The incubation temperature was set to 30°C. Bacteria were pre-cultivated in anoxic medium with ferric citrate as electron acceptor as described above. Before inoculation into BES, cells were washed in synthetic medium without electron acceptor and then transferred to BES filled with wastewater. The final OD_600_ was adjusted to 0.02 for each strain in mixed-culture experiments and OD_600_ 0.1 for single-species experiments. In batch-mode experiments, the medium was fully exchanged every 7 days. In continuous-mode experiments, BES were initially kept in batch-mode for 9 days with synthetic medium. Subsequently, wastewater was pumped through the reactor with a multi-channel peristaltic pump (IPC, Ismatec; Germany) and a flowrate of 0.015 ml/min [hydraulic retention time (HRT) 12.5 days]. At day 23, the flowrate was increased continuously to 0.293 ml/min (HRT 6.5 days). From day 39 on, the flowrate was successively decreased to 0.015 ml/min within 10 days and then held constant until the end of the experiment. The pH of the reactors was monitored daily and adjusted to 7.2 using sodium hydroxide, if necessary. To illustrate the conduct of the continuous-mode experiments, the variations of the pump rate are indicated in **Figure 4**.

#### Sample Preparation and Electrochemical Measurements

Samples were taken at least every 2–3 days and stored at −20°C prior to subsequent analysis. At the end of each experiment, the anodes were removed and divided into three parts that were further analyzed.

Coulombic efficiency (CE) was calculated by dividing the detected number of electrons transferred to the anode by a theoretically calculated value. For this, it was assumed that each molecule of organic carbon has a redox state of 0 [following the simplified formula of organic carbon (CH_2_O)_n_] and by this, the oxidation of one molecule of organic carbon to CO_2_ releases four electrons.

### RNA Isolation, RNA Sequencing, and 16S rRNA Gene Sequencing

Prior to RNA isolation, the anode slices and liquid cultures were stored at −20°C in LifeGuard™ Soil Preservation Solution according to the manufacturer‘s instructions (MoBio; USA). After thawing, the cells were loosened gently from the anode surface using a cell mill (MM400, Retsch; Germany) for 30 min at 8 Hz. RNA was isolated with the RNA Power®Total RNA Isolation Kit (Qiagen; Germany) according to the manufacturer's instructions. DNA was removed with the DNA-*free*™ Kit (Thermo Fischer; USA) at 37°C. Sequencing libraries were prepared from 400 ng of total RNA samples following the TruSeq stranded RNA protocol (Illumina; USA, without purification). Sequencing was performed on a HiSeq1500 using SBS v3 kits (Illumina) generating paired-end reads of 2 × 50 nucleotides. Cluster detection and base calling were performed using RTA v1.13 (Illumina). The read quality was evaluated with CASAVA v1.8.1 (Illumina). 255 million reads were generated via sequencing with 93% of them having a quality Phred score of Q30 or more.

For the isolation of 16S rRNA, 1 ml of a liquid culture was harvested and the pellet was resuspended in 0.9% NaCl and heated for 5 min at 90°C. The 16S rRNA genes were amplified using this suspension as template and the primers Bak27F and BakUniversal1492R (Supplementary Table 1) Sequencing was carried out by GATC (GATC Biotech AG, Konstanz).

### RNA-Sequence Data Analysis

The sequence data of the continuous-mode and the planktonic adaptation experiments were mapped against the genome sequence of *G. sulfurreducens* (NCBI-Acc.No. NC_002939.5) using bowtie2 (Langmead and Salzberg, 2012) and sorted by position on the chromosome with samtools (Li et al., 2009). The absolute gene expression was calculated as reads per gene identifying the number of reads compared to the annotated gene loci using htseq (Anders et al., 2015). The reads were normalized and the differential expression was calculated with R package DESeq2 subsequently (Love et al., 2014). Additionally, the sequence data of the continuous-mode experiment (setup 2) were aligned to 51 selected protein sequences using RAPsearch2 (Zhao et al., 2012) to identify possible metabolic pathways of the isolates. The results were filtered for hits with >50% identity and >9 amino acids in length. The hits were summed up for each protein and divided by the total number of hits. For RPM-normalization (reads per million) the results were multiplied by 10^6^. For evaluation, reads with RPM > 300 were analyzed. All raw reads of the sequencing that were retrieved for this study are publicly available through NCBI BioProject PRJNA475466 under SRA accession: SAMN09487749, SAMN09487633.

### DNA Isolation and qPCR

DNA isolation was performed using the innuPREP Stool DNA kit (Analytic Jena; Germany) according to the manufacturer's instructions with minor modifications. Five milliliters of SLS buffer and 1.5 g glass beads (0.1–0.25 mm, Retsch; Germany) were added to the anode slices and the samples were placed in a cell mill (MM400, Retsch; Germany) for 7 min at 30 Hz. After an incubation at 95°C for 15 min, 2 × 1 ml of each sample were transferred into a new reaction tube and centrifuged at 8,000 g for 2 min. Six hundred and fifty microliters of the supernatant were used according to the manufacturer's protocol. Quantitative PCR was conducted as described in Dolch et al. (2015) using SsoAdvanced™ Universal SYBR® Green Supermix and primers G.s._barcoding_qPCR_for and G.s._barcoding_qPCR_rev (Supplementary Table 1). DNA concentration was normalized to cell numbers based on standard curves generated from *G. sulfurreducens*_bc_ cells. For this, cells were counted in a counting chamber (improved Neubauer; Germany) prior to DNA isolation.

### Fluorescent *in situ* Hybridization (FISH)

FISH experiments were carried out according to Dolch et al. (2014). Probes and helper oligonucleotides are listed in Supplementary Table 1. Image acquisition was conducted with a Leica DM 5500 B microscope using a 63 × water immersion lens and a DFC 300 FX digital color camera (Leica; Germany). The filter sets L5 (excitation filter 480/40 and suppression filter 527/30), Y3 (545/30 and 610/75), Y5 (620/60 and 700/75), and A4 (360/40 and 470/40) were used for the fluorescent dyes FITC, Cy3, Cy5, and DAPI.

### Analytical Measurements

Samples were taken every 2–3 days and all samples were filtered through a 0.2 μM filter prior to analysis.

For growth experiments of the isolates, Fe(III)-reduction was determined spectrophotometrically using the ferrozine assay described elsewhere (Stookey, 1970).

Total nitrogen (TN) and total organic carbon (TOC) were determined using a TOC-analyzer (Multi N/C 2100 S Analytic Jena, Germany) and ${\text{NO}}_{2}^{-}$ and ${\text{NH}}_{4}^{+}$ were quantified with a spectral photometer DR3900 (Hach Lange, Germany) and cuvette tests (Grießmeier et al., 2017).

Analysis of acetate concentration was performed via HPLC (UltiMate3000; Thermo Fisher Scientific, USA) using an Aminex HPX-87H column as described elsewhere (Sturm-Richter et al., 2015).

The conductivity of the wastewater was measured using an HI 99300 EC/TDS meter (HANNA Instruments, Germany).

## Results

The aim of this study was to establish an efficient anode community for the elimination of organic carbon sources from an industrial wastewater stream coupled to the simultaneous production of high current densities. We reached this aim via isolation of exoelectrogenic members of the natural community as well as with the adaptation of the model organism *G. sulfurreducens*_bc_.

### Isolation Experiments and 16S rDNA Gene Sequencing

For the isolation of exoelectrogenic bacteria from industrial wastewater, ferric citrate was used as a surrogate for an insoluble electron acceptor as it was shown that it is reduced at the cell surface of model organisms for extracellular electron transfer (Lovley et al., 2004). With his strategy, four different strains could be isolated: *Clostridium* sp. isolate I, *Clostridium* sp. isolate II, an *E. coli* strain (isolate III) and *Paenibacillus* sp. (isolate IV; Table 1).

**Table 1:** Isolated organisms from industrial wastewater.

**Strain**	**Phylogenetic classification^a^**	**Identity%^a^**	**fragment length (bp)^b^**	**Accession no.^a^**
1	*Clostridium sartagoforme*	99	1,357	KU950266.1
2	*Clostridium butyricum*	99	1,379	CP013239.1
3	*Escherichia coli*	99	1,389	HG428755.1
4	*Paenibacillus phoenicis*	99	1,371	NR108292.1

a *Results of the phylogenetic classification, identity (%) and accession no. of the closest database match via NCBI nucleotide blast*.

b *Fragment length (bp) of the aligned sequences*.

**Figure 1:**
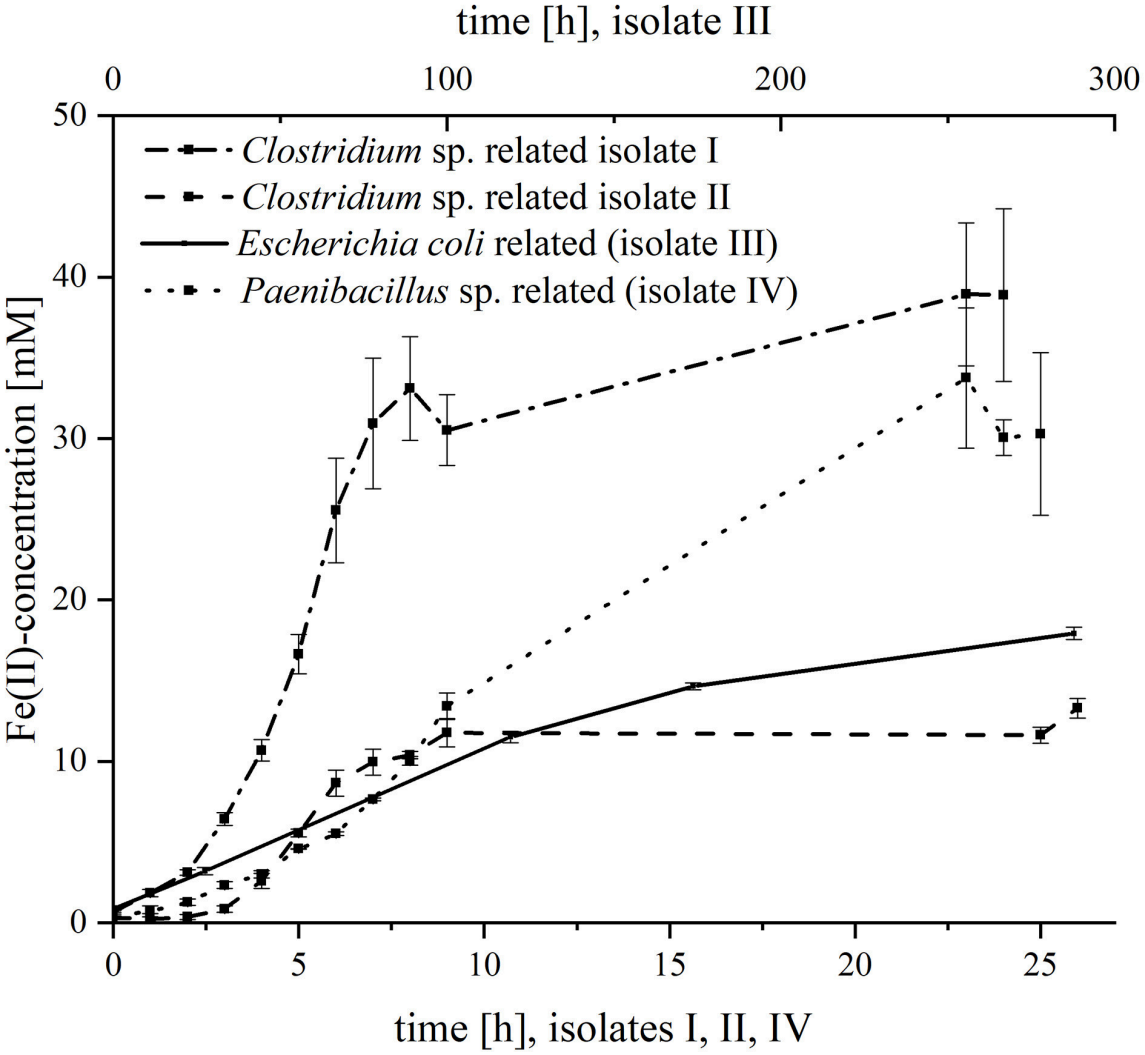
Fe(III)-reduction of the four isolates. The figure shows the Fe(II)-concentration [mM] over time [h]. The lower x-axis is assigned to *Clostridium* sp. isolate I, *Clostridium* sp. isolate II and *Paenibacillus* sp. (isolate III); and the upper x-axis is assigned to *Escherichia coli* (isolate IV). Error bars indicate standard deviation of independent triplicates.

Subsequently, the four organisms were analyzed separately regarding their kinetics in ferric citrate reduction (Figure 1). All strains were able to reduce iron, but with variable reduction rates and final ferrous iron concentrations. The fastest iron reduction was recorded for *Clostridium* sp. isolate I (most closely related to *C. sartagoforme*) with maximum rates of 1.6 mM Fe^2+^/h. Interestingly, *Clostridium* sp. isolate II (most closely related to *C. butyricum*) reduced ferric citrate with lower rates (0.6 mM Fe^2+^/h) and the final ferrous iron concentration was with 13.3 mM more than 3-fold lower compared to isolate I with 38.9 mM Fe^2+^. With 30.3 mM, the *P. phoenicis* related isolate IV reduced ferric iron with roughly equal amounts compared to isolate II, but the reduction rate was considerably slower (1.3 mM Fe^2+^/h). The slowest reduction rates (0.1 mM Fe^2+^ h^−1^) and the lowest final ferrous iron concentration (3.0 mM) were measured with the *E. coli* related isolate III.

### Wastewater Treatment and Electricity Production in BES

The wastewater used in this study was originated from a chemical park and had TN content of 524 mg/L and a TOC content of 1.99 g/L. Fifty-seven percent of the TOC could be related to acetate, which has a concentration of 47.2 mM. Moreover, the water contained cellulose fragments and potentially other carbon compounds that could not be specified in this study. The pH was 7.8 and the conductivity 6.5 mS. The wastewater contains nitrate and ammonium in a concentration of 0.3 and 17.6 mM, respectively.

**Figure 2:**
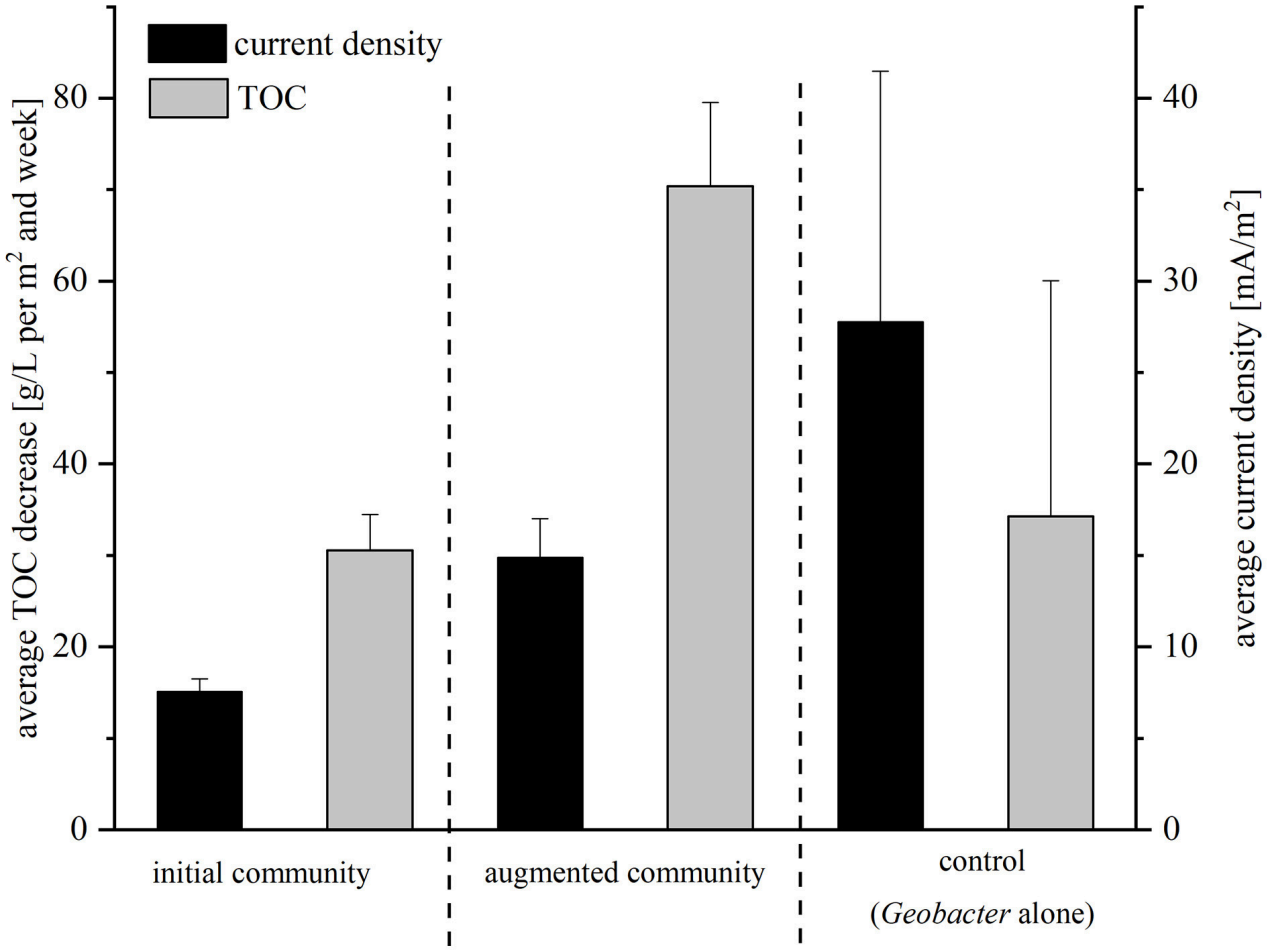
Average TOC decrease and average current density for all three setups of the batch mode experiment in comparison. Current density was recorded in mA/m^2^ and is indicated with black bars; TOC was measured in g/L per m^2^ and week and is depicted in gray bars. Error bars indicate standard deviation of independent triplicates.

The performance of the isolated strains was analyzed in BES with this wastewater under batch-mode conditions under different conditions. First, the electrochemical activity of a coculture of the four isolated strains was analyzed (we will refer to this community from now on as *initial community*); whereas in the second setup the lab strain *G. sulfurreducens*_bc_ complemented the community (the mixture of isolates and *G. sulfurreducens*_bc_ will be referred to as *augmented community* from here on). The average current density measured over a period of 21 days was 7.6 mA/m^2^ for the initial community and 14.9 mA/m^2^ for the augmented community. Hence, addition of the lab strain led to 2-fold increased current densities (Figure 2). During this time, 30.6 g TOC per m^2^ and week were oxidized by the isolates alone and 70.4 g TOC per m^2^ and week in coculture with *Geobacter* (Figure 2). The calculated CEs were extremely low in both experiments with 1.6 and 1.3%, respectively. Based on these two experiments with low performance concerning power output and TOC degradation, a control experiment with *G. sulfurreducens*_bc_ alone was performed. The average current density was 27.8 mA/m^2^ and 34.3 g TOC per m^2^ and week were degraded in this setup (6% of total TOC) (Figure 2). With 4.8%, CE was slightly higher compared to the cocultivations.

**Figure 3:**
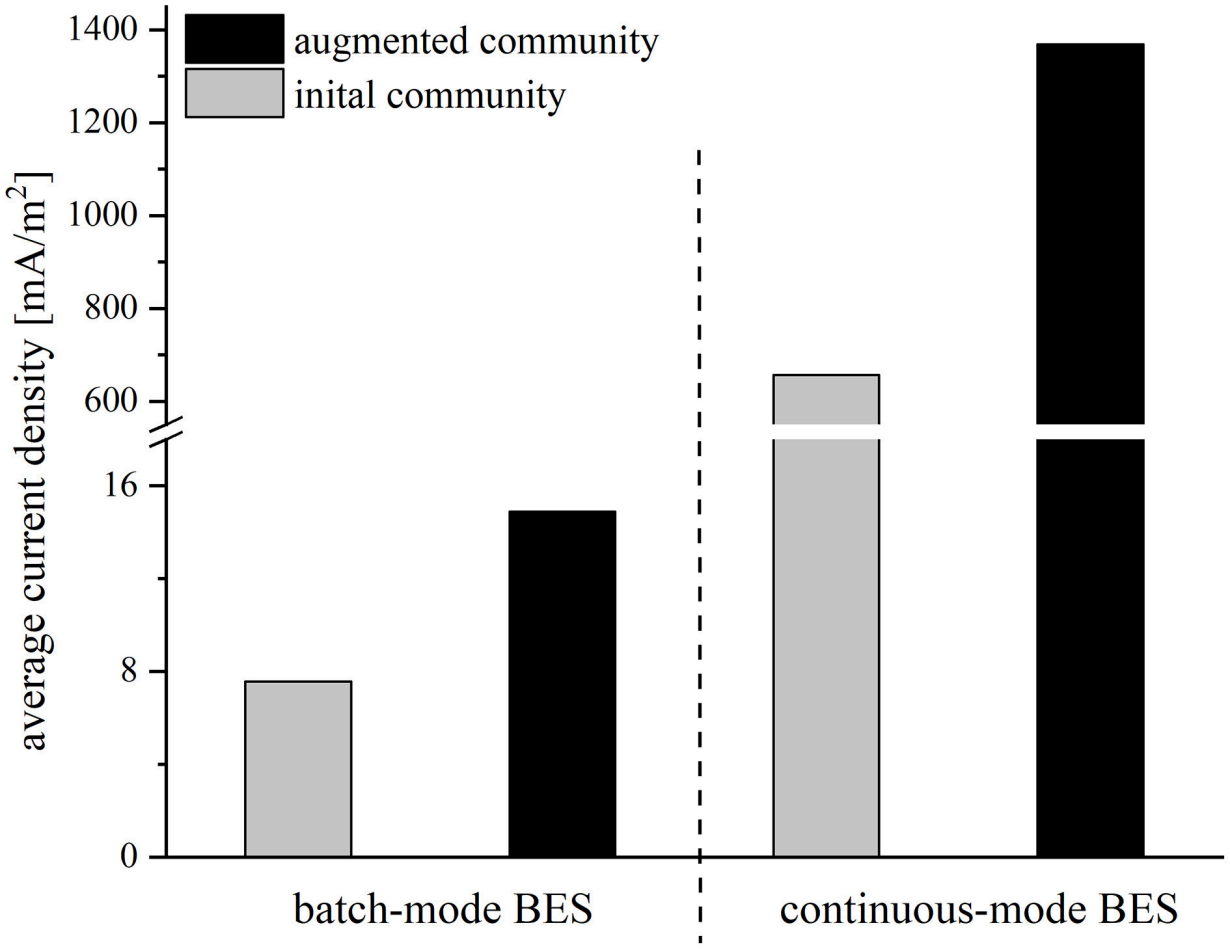
Average current density [mA/m^2^] of the initial community (gray bars) and the augmented community (black bars) in batch mode and continuous mode reactors operated with wastewater.

The performance of all strains was rather low, possibly due to the abrupt change of medium from synthetic medium to wastewater. To facilitate a smoother transition between the preculture and the BES medium and an adaptation of the strains, the reactor conditions were changed from batch-mode to continuous-mode. The experiments were started with an initial batch period with synthetic medium to allow for an attachment of the cells to the anode. Then, the synthetic medium was slowly replaced by wastewater with a flowrate of 0.015 ml/min, so that the strains could gradually adapt to wastewater conditions in order to achieve a better performance compared to the batch-mode reactors. After 21 days, when the synthetic medium was fully replaced by wastewater, the average current density was 657.0 mA/m^2^ for the initial and 1368.3 mA/m^2^ for the augmented community. Again, the addition of *Geobacter* to the community doubled the average current density. Compared to the batch-mode systems the average current density in the adapted cocultures increased 82-fold (initial community) and 91-fold (augmented community), respectively (Figure 3).

**Figure 4:**
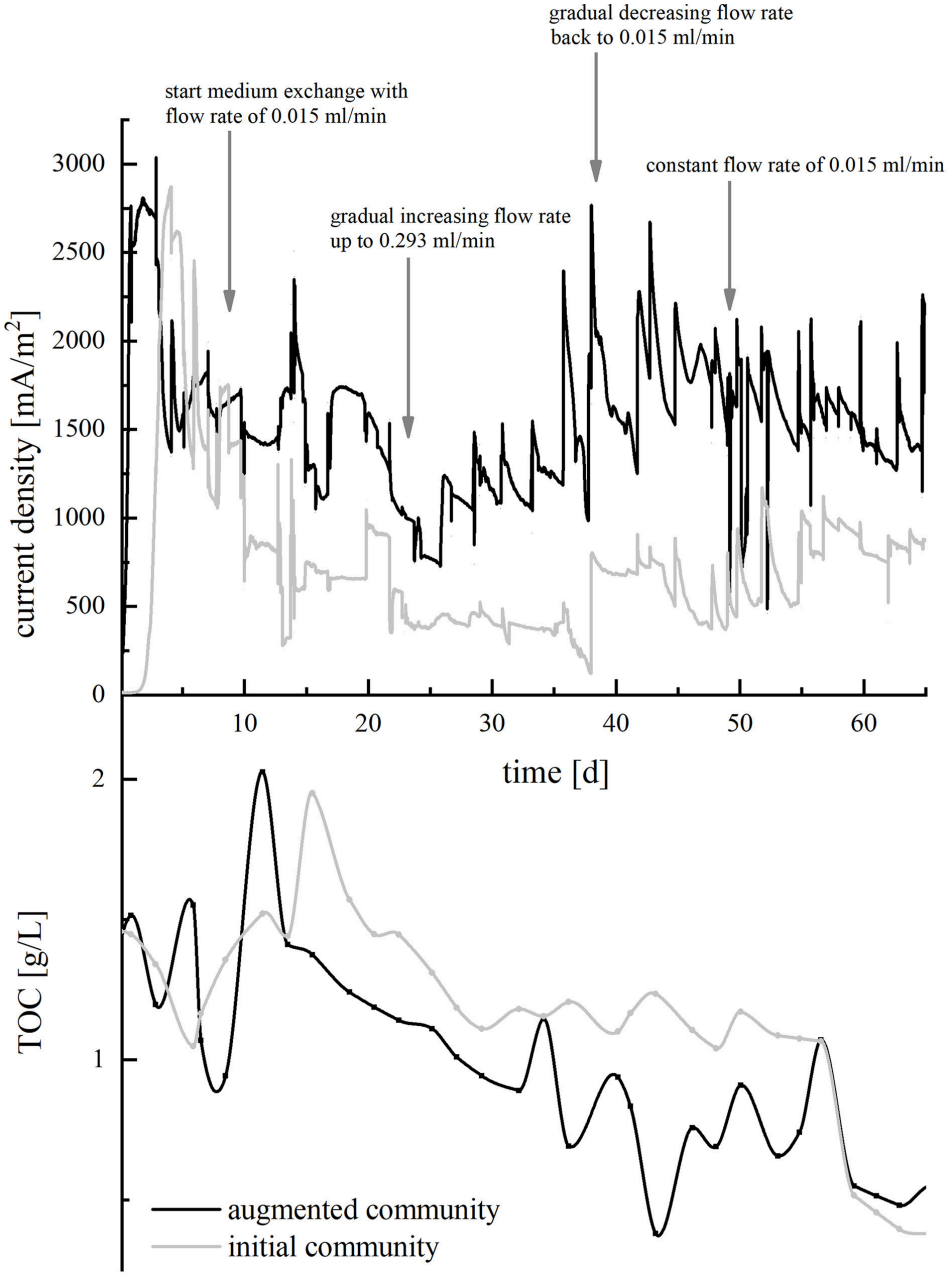
Course of current density (upper half, in mA/m^2^) and TOC concentration (lower half, in g/L) in the continuous-mode BES over a period of 65 days. BES with the initial community is depicted in gray, and BES with the augmented community is depicted in black. Arrows indicate the time points, when the pump rate of the medium was varied. For the current density graph, curve progression was smoothed using the b-spline algorithm.

Now, HRT of the wastewater was decreased in order to further evaluate the performance of the microbial communities regarding TOC removal and CE. In BES inoculated with the isolates only, increasing flow rates resulted in a stepwise decrease of current density, whereas current production increased in BES containing the augmented community. Returning to a longer HRT from day 39 on could reverse this effect (Figure 4, top). In both setups, TOC removal proceeded relatively uniformly and decreased successively from around 1.5 g/L to 0.5 g/l until day 59, when a blocked tubing inhibited in the wastewater inflow and resulted in an interrupted TOC removal. Until day 59, 29.9% TOC were degraded by the initial and 72.9% by the augmented community, respectively (Figure 4, bottom). To be comparable with the data obtained from the batch-mode reactors, CE was calculated from TOC consumption during the time of the initial slow flowrate (day 18 until 22) and yielded in 23.8% for the adapted initial community and 54.9% for the adapted augmented community. Accordingly, adaptation of the organisms to the wastewater improved the CE by a factor of 14.9 (initial community) and 42.3 (augmented community), respectively.

### Organisms on the Anode and Cell Number of *G. sulfurreducens*_bc_

FISH analysis of the anode communities was conducted with the aim to correlate current density with biomass content. In these experiments, the augmented communities from batch and continuous experiments were compared and FISH images indicate a drastically increased biofilm thickness on anodes after gradual adaptation to the wastewater. Besides that, the images reveal that the biofilm consists mainly of *G. sulfurreducens*_bc_ cells and only a few *E. coli* cells, whereas the *Clostridium* and *Paenibacillus* isolates dominate the planktonic phase (Supplementary Figure 1). Moreover, the cell number of *G. sulfurreducens*_bc_ in the anode biofilms was quantified via qPCR. The analysis revealed a 5,000-fold increase of the cell number of *G. sulfurreducens*_bc_ from batch- to continuous-mode (3.16 × 10^6^ vs. 1.5 × 10^10^ cells, respectively). However, this enormous cell growth only led to a 91-fold increase in current density (Figure 3).

### Adaptation Experiments With *G. sulfurreducens*_bc_ in Wastewater

A transcriptomic study was conducted to understand the mechanism of adaptation and consequently get potential evidence for the reason of growth limitation. The aim was to compare non-adapted and adapted *G. sulfurreducens*_bc_ cells from batch cultures with fumarate as electron acceptor with cells from the anode community at the end of the continuously conducted experiment described before. If there was a specific adaptation of the strain toward wastewater as medium, this mechanism of adaptation should become clear under both conditions, single species planktonic growth with fumarate and multispecies biofilm growth on anodes. Of note, due to the low number of cells on the anodes at the beginning of the experiment, it was not possible to compare non-adapted and adapted cells that both originate from anodes.

**Figure 5:**
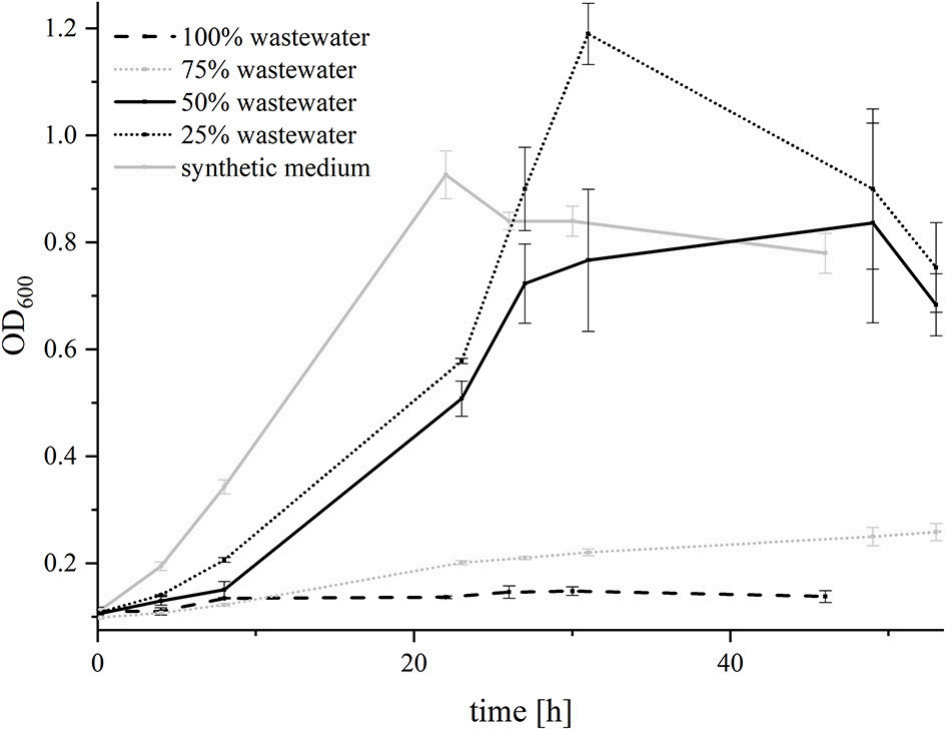
Growth of *G. sulfurreducens*_bc_ with different wastewater concentrations. Cell growth was measured photometrically and is depicted for 100% wastewater (dashed black line), 75% wastewater (dotted gray line), 50% wastewater (solid black line), 25% wastewater (dotted black line) and synthetic medium (solid gray line). Forty millimolars of fumarate were added as electron acceptor. The acetate concentration was adjusted to 21 mM in all experiments. Error bars indicate standard deviation of independent triplicates.

*G. sulfurreducens*_bc_ was not able to grow in 100% wastewater and barely grew in 75% wastewater. In medium with 50 and 25% wastewater, it showed similar growth characteristics as in the synthetic medium alone (Figure 5). Hence, the adaptation experiment was conducted with 75% wastewater to analyze if the strain was able to improve its growth over 6 generations of adaptation (AG—adaptation generation) by inoculating the cells every week in a new batch of medium consisting out of 75% wastewater and 25% synthetic medium with fumarate and acetate. Interestingly, growth of *G. sulfurreducens*_bc_ was affected by the addition of wastewater more strongly after the first transfer, as the doubling time increased from 20.2 h in AG1 to 56.7 h in AG 2. In the following transfers (AG 3 to AG 6), the doubling decreased from 37 to 27.1 h, indicating that there is a detectable adaptation of the organisms over time.

The transcriptomic analysis of the fumarate grown cells and the metatranscriptomic analysis of the biofilm grown cells revealed clear differences in expression levels of several genes encoding proteins involved in extracellular electron transport, central metabolism (TCA cycle and acetate oxidation), stress response and membrane transport. In our analysis, we searched for genes that displayed similar expression patterns (log2fold change ± 2) if the adapted cells or the continuous mode biofilm were compared to the non-adapted planktonic culture (Supplementary Table 2). In other words, it was our hypothesis that similar adaptation events lead to more robust growth under planktonic as well as under biofilm growth conditions. In both cases, genes encoding the NADH dehydrogenase I and enzymes of the citric acid cycle were clearly upregulated, indicating a generally increased metabolic rate. Besides several cytochromes, genes for hypothetical proteins and prophage genes, we also observed an upregulation of transporters and exopolysaccharide synthesis genes. In contrast to 90 genes being upregulated compared to the non-adapted strain, only six genes were downregulated under both conditions. Four of these encode hypothetical proteins, while one gene encodes a putative response regulator and the other one a potential nickel transport protein.

### Identification of Key Enzymes for Possible Metabolic Pathways in the Continuously Operated BES

A further aim was to identify possible metabolic pathways of all the isolates and *G. sulfurreducens*_bc_ in the continuously operated BES. The transcriptomic data showed transcripts indicating the presence of the following metabolic pathways: cellulose degradation, Embden-Meyerhof pathway, mixed acid fermentation, acetate biosynthesis, butyric acid fermentation, Wood-Ljungdahl pathway, Bifid-Shunt, butandiol biosynthesis, lactate biosynthesis, pyruvate oxidation, different types of anaerobic respiration and Stickland fermentation (Fuchs, 2014). Table 2 shows the identified key enzymes which were assigned to the mentioned pathways. Moreover, the respective reads were assigned to potential genera. This assignment indicates growth of the Clostridia using cellulose and amino acids as carbon and energy source conducting Stickland and butyric acid fermentation. Moreover, a sub-fraction of the Clostridia seems to thrive autotrophically as transcripts of the Wood-Ljungdahl pathway were detectable. *E. coli* seems to grow using mixed acid fermentation and by respiring on available electron acceptors. *Paenibacillus* transcripts of central metabolic pathways mainly could be assigned to cellulose degradation and bifid-shunt based fermentation. Moreover, some transcripts of central metabolic pathways were most closely related to organisms belonging to the genera *Streptococcus* and *Campylobacter*, which were not part of the cultivated community. As we have not sequenced the genomes of the isolated strains and used available sequences of closely related organisms, it is possible that this is due to horizontal gene transfer.

**Table 2:** Data of the metatranscriptomic analysis.

**Protein (GI)**	**RPM**	**Potential metabolic pathway**
Cellulase (510834545)^a^	1,139	Cellulose degradation
Cellobiose phosphorylase (573579109)^c^	726	Cellulose degradation
6-phosphofructokinase I (85676144)^b^	960	Embden-Mayerhof pathway
Pyruvate formate-lyase protein (1024797169)^d^	5,124	Mixed acid fermentation
Acetate kinase (190909529)^b^	390	Mixed acid fermentation
Acetate kinase (764112765)^c^	726	Acetate biosynthesis
Formate dehydrogenase H, selenocysteine-containing (930359049)^e^	344,286	Mixed acid fermentation
Formate dehydrogenase, alpha subunit (953087628)^a^	3,677	Mixed acid fermentation
Pyruvate ferredoxin oxidoreductase (923387097)^a^	976	Butyric acid fermentation
Hydrogenase (160624920)^a^	368	Butyric acid fermentation
Carbon monoxide dehydrogenase (300437026)^a^	839	Wood-Ljungdahl pathway
CO dehydrogenase/acetyl-CoA synthase, acetyl-CoA synthase subunit (308066779)^a^	305	Wood-Ljungdahl pathway
Phosphoketolase (305856304)^c^	599	Bifid-Shunt
Acetolactate synthase (917007469)^c^	9,680	Butandiol biosynthesis
Phosphate acetyltransferase (738695293)^c^	308	Acetate biosynthesis
L-lactate-dehydrogenase (336299116)^c^	358,753	Lactate biosynthesis
Pyruvate decarboxylase (941132041)^a^	601	Pyruvate oxidation
DMSO reductase (612260286)^b^	226,761	Anaerobic respiration
L-lactate dehydrogenase (974705824)^b^	775	Anaerobic respiration
Nitrite reductase (751402387)^b^	2,374	Anaerobic respiration
Nitrate reductase (937299854)^b^	4,645	Anaerobic respiration
D-proline reductase (800899476)^a^	16,861	Stickland fermentation
(R)-2-hydroxyisocaproyl-CoA dehydratase alpha subunit (75361392)^a^	4,399	Stickland fermentation

*RNA reads were mapped to 51 protein sequences corresponding to possible metabolic key enzymes. Gene expression level is depicted in reads per million base pairs (RPM). Key enzymes were assigned to possible metabolic pathways*.

a *>50% identity with a sequence of a species of the genus Clostridium*.

b *>50% identity with a sequence of E. coli*.

c *>50% identity with a sequence of a species of the genus Paenibacillus*.

d *>50% identity with a sequence of a species of the genus Streptococcus*.

e *>50% identity with a sequence of a species of the genus Campylobacter*.

## Discussion

This study was based on the hypothesis that an acetate rich wastewater could be an excellent medium for current production and simultaneous carbon elimination in a bioelectrochemical system using *G. sulfurreducens* as main biocatalyst. Furthermore, we hypothesized that the addition of wastewater adapted strains could further accelerate the process and make other carbon sources available.

The ability to thrive using Fe(III) or an anode as its surrogate as respiratory electron acceptor seems to be not uncommon in microbial taxa. Besides *Geobacter* and *Shewanella* species (Lovley et al., 2004; Mahadevan et al., 2006; von Canstein et al., 2008) numerous other species seem to have this ability although it is often not fully elucidated how the electron transfer functions in detail (Roden and Lovley, 1993; Lonergan et al., 1996). *E. coli* or *Clostridium* strains were initially not considered to reduce ferric iron but this work and others have provided evidence, that Fe(III) can not only be a primary respiratory electron acceptor but also a secondary electron acceptor under fermentative conditions. For example, *E. coli, C. butyricum* EG3, and *C. beijernickii* can use Fe(III) as electron sink, which presumably gives the organisms an energetic advantage (Emde et al., 1989; Lovley, 1991; Dobbin et al., 1999; Park et al., 2001). Hence, we hypothesize that the same is true for the here presented isolates. The exact mechanisms for using iron as electron sink have not been identified yet. Dobbin and colleagues were able to identify a NAD(P)H-dependent Fe(III) reductase activity localized to the membrane of *C. beijernickii* (Dobbin et al., 1999). Coupling the oxidation of NAD(P)H to the reduction of Fe(III) could result in a higher availability of NAD(P)^+^ which accelerates the metabolism and allows to use more of the carbon source for ATP production and less for balancing of the redox status (Lovley and Phillips, 1986; Park et al., 2001).

The BES batch-mode experiments revealed that the cultivation of the isolates and *G. sulfurreducens*_bc_ together had an additive effect, which suggests that the organisms use different carbon sources of the wastewater. The isolates alone catalyzed roughly 50% of the current production and carbon elimination compared to the augmented community composed of the wastewater isolates and *G. sulfurreducens*_bc_ together. Still, the performance was rather limited and CE extremely low. The latter suggests that other products were formed as electron sink like for instance hydrogen. Moreover, we cannot exclude low oxygen contaminations of the system, which could have partly sustained carbon elimination, too. Strain adaptation turned out to be a suitable solution to increase the current density. The wastewater was pumped with a low flowrate of 0.015 ml/min (HRT = 12.5 days) into the medium-filled BES to give the adaptation process sufficient time. The success of the process is mirrored in the 71- and 88-fold (BES with the initial community 7.6–540.4 mA/m^2^, BES with the augmented community 14.9–1317.3 mA/m^2^) increase in current density compared to the batch-mode experiments. By adding the lab strain *G. sulfurreducens*_bc_ to the system, the average current density could be more than doubled from 540.4 to 1317.3 mA/m^2^. Moreover, adaption of the cells to the wastewater increased the calculated CE for both setups to 23.8 and 54.9%, respectively and at least the latter value could be sufficient for a potential application. Of note, the 88-fold increase in current density was accompanied by 5,000-fold more *Geobacter* cells compared to the batch-mode experiment. This suggests that the activity of the majority of the community is limited by the electron transfer to the electrode surface. Hence, further design of the BES and the biofilm could promise by far higher current densities than we have measured so far.

The increased current densities that were achieved in the continuous-mode experiments seemed to be a result of strain adaptation. We tried to mimic this adaptation by a planktonic growth experiment with *G. sulfurreducens*_bc_ in 75% wastewater. However, over 5 weeks in 75% wastewater the growth of the strain improved only slightly. So far, we cannot say why growth was more affected after the second transfer in wastewater. A potential reason why the adaptation was more efficient in the BES could be that *G. sulfurreducens*_bc_ was forced to grow as a biofilm, which seems to be a more robust form of growth *per se*. Nevertheless, we could detect genes that were up- or downregulated in both adaptation experiments compared to the initial culture. Upregulation of gene clusters for the NADH dehydrogenase display the reason for higher current densities but not for the adaptation. The observed upregulation of transporters and exopolysaccharide synthesis genes might indicate that a growth-inhibiting factor is constantly exported and/or increased extracellular matrix production might be used as a diffusion barrier or biosorbent for potentially growth inhibiting factors.

**Figure 6:**
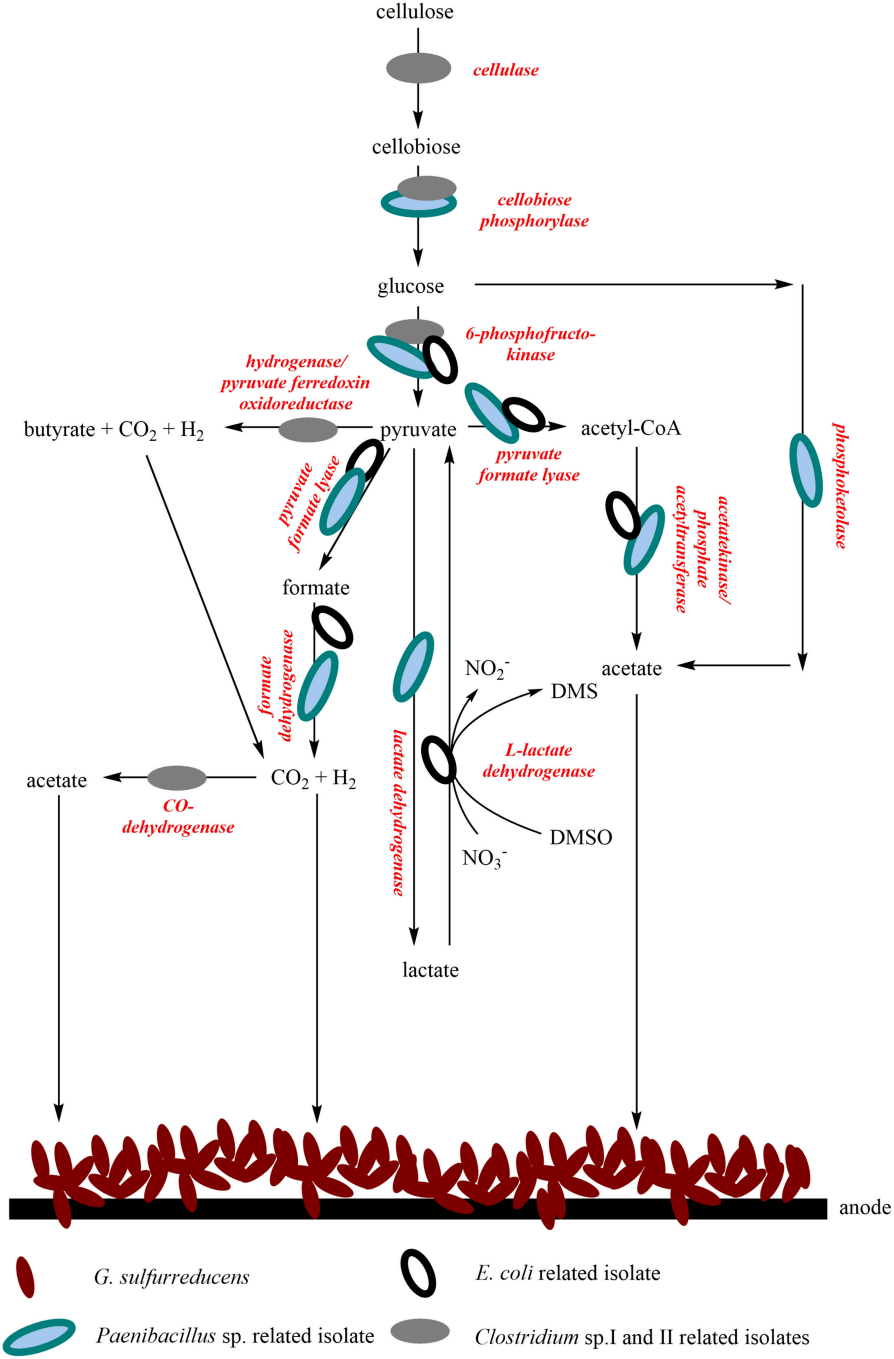
Possible syntrophic interactions of the four isolates and the lab strain *G. sulfurreducens*_bc_ in the continuous-mode reactor operated with wastewater. *G. sulfurreducens*_bc_ transfers electrons directly to the anode either via a trans-outer membrane porin cytochrome complex, consisting of a periplasmic *c*-type cytochrome, a porin-like protein, and a reductase in the outer membrane; or via conductive pili, so called nanowires (Simonte et al., 2017). For the wastewater isolates, the mechanism of extracellular electron transfer has not been identified so far.

A comparison of the isolated RNA with possible key enzymes involved in central metabolism revealed the function of the isolates in the bioelectrochemical system and suggests a syntrophic interaction. It can be assumed that the wastewater contains cellulose, as this is one of the substances used in the chemical park. Cellulose can serve as a substrate for Clostridia and can be converted into glucose via cellobiose (Demain et al., 2005). Cellobiose can also be metabolized by *Paenibacillus polymyxa* (Adlakha et al., 2015) and it could be assumed that the same might be true for the *Paenibacillus* isolate analyzed in this study. Most probably, glycolysis can be conducted by all four isolates. Pyruvate represents an intermediate of glucose oxidation and can also be further metabolized in several ways. By mixed acid fermentation, *E. coli* can generate a series of fermentation products, like acetate, CO_2_ or H_2_. Acetate can also be produced by several other processes, for example the so-called bifid shunt of *P. polymyxa*, a glycolytic bypass described by Adlakha et al. (2015) or the Wood-Ljungdahl pathway, which is carried out by *Clostridium* strains (Ragsdale and Pierce, 2008). The isolated Clostridia could also conduct butyric acid fermentation, which would lead to the production of CO_2_ or H_2_. Acetate, H_2_ and CO_2_ are substrates for *G. sulfurreducens*. It seems as if *E. coli* cells express nitrate and DMSO reductase genes. Nevertheless, we could not detect DMSO in the wastewater so far. In summary, we suggest that the isolates metabolize complex organic substrates to organic acids, H_2_ and CO_2_, which can be used by *G. sulfurreducens*_bc_ and some of the isolates themselves. Figure 6 summarizes possible syntrophic interactions of the isolates and *G. sulfurreducens*.

## Conclusion

In this study, we report on the efficient isolation of ferric iron reducing microorganisms from an industrial wastewater and their application as coculture in BES. Continuous-mode BES revealed strongly increased current densities compared to batch-mode experiments, presumably due to strain adaptation. Moreover, our results illustrate that industrial wastewater can provide a sufficient medium for *G. sulfurreducens* and augmentation of the initial coculture with the laboratory strain leads to much more efficient elimination of organic carbon from the wastewater and drastically increased current density and CEs. For engineering applications of BES in wastewater treatment this emphasizes several things: iron-reducing isolates from the original wastewater are promising candidates for carbon removal and current production, mixed bacteria communities are advantageous compared to single strain cultures and slow adaptation of exoelectrogenic laboratory strains to wastewater can be a key factor for their efficient application.

In future experiments, we will try to isolate the specific adaptation targets in *G. sulfurreducens* and will build a library of genomic and transcriptomic adaptations that could increase the performance of *Geobacter* strains in wastewater-driven BES.

## Author Contributions

SB carried out the experiment. TK wrote the manuscript with support from KS-R. AD analyzed the transcriptomic data. JG supervised the project.

### Conflict of Interest Statement

The authors declare that the research was conducted in the absence of any commercial or financial relationships that could be construed as a potential conflict of interest.
