# Supplementary material for: Efficient Bioelectrochemical Conversion of Industrial Wastewater by Specific Strain Isolation and Community Adaptation
Source: Front Bioeng Biotechnol. 2019 Feb 19;7:23. doi: 10.3389/fbioe.2019.00023 (PMC6389598; doi:10.3389/fbioe.2019.00023)
Supplement: Supplementary file 1 [file Data_Sheet_1.docx]

**Supplementary material**

*Supplementary Table 1: Sequences of primers used in this work; probes and helper oligonucleotides for FISH.*

| primer pairs | sequence 5´→ 3´ | target |  |
| --- | --- | --- | --- |
| 27F  Universal1492R | GAGTTTGATCCTGGCTCA  GGTTACCTTGTTACGACTT | Bacteria 16S  Bacteria 16S |  |
| G.s. barcoding qPCR for  G.s. barcoding qPCR rev | CGGTTCTATCGACCTACC  CTGCTTGATGAACGAGAG | *G. sulfurreducens* (Barcode)  *G. sulfurreducens* (Barcode) |  |
| G.s. Genom for  G.s. Genom rev | AACGACCTGCTCAGCCTG  TTTCAGGGCCTCATCCTGG | *G. sulfurreducens*  *G. sulfurreducens* |  |
| ^1^Ent/16S rRNA | CCCCCWCTTTGGTCTTGC | Enterobacteriaceae |  |
| ^2^LGC/16S rRNA | TCACGCGGCGTTGCTC | Firmicutes |  |
| ^3^Geo_2/16S rRNA | GAAGACAGGAGGCCCGAAA | *G. sulfurreducens* |  |
| ^3^HGeo_2.1/helper oligo 1 Geo_2 | GTCCCCCCCTTTTCCCGCAAGA | *G. sulfurreducens* |  |
| ^3^HGeo_2.2/helper oligo 2 Geo_2 | CTAATGGTACGCGGACTCATCC | *G. sulfurreducens* |  |
| ^3^EUB338-1/16S rRNA | GGTTACCTTGTTACGACTT | Eubacteria |  |

^1^ (Kempf *et al.*, 2000)

^2^ (Küsel *et al*., 1999)

^3^ (Richter *et al*., 2007)

*Supplementary Table 2: FoldChange of expressed genes of TCA-cycle and acetate-oxidation of AG 6 vs. AG 1, C-MR vs. AG 6 and C-MR vs. AG1 in metatranscriptomic analysis. C-MR abbreviates continuous-mode reactor; AG adaptation generation)*

| Locus ID | gene | annotation | FoldChange AG 6 vs. AG 1 | FoldChange C-MR vs. AG 6 | FoldChange C-MR vs. AG 1 |
| --- | --- | --- | --- | --- | --- |
| GSU0097 | *por* | pyruvate ferredoxin oxidoreductase | 1,768 | 4,245 | 7,505 |
| GSU0174  GSU0490 | *ato-2*  *ato-1* | acetyl-CoA transferase | 4,121  2,602 | 3,476  9,280 | 14,326  24,147 |
| GSU0994 | *fumB* | fumarate hydratase | 4,286 | 3,732 | 15,958 |
| GSU1058  GSU1059 | *sucC*  *sucD* | succinyl-CoA synthetase | 2,261  3,966 | 1,359  -1,190 | 3,073  3,332 |
| GSU1106 | *gltA* | citrate synthase | 3,097 | 7,698 | 23,841 |
| GSU1176  GSU1177  GSU1178 | *frdC*  *frdA*  *frdB* | fumarate reductase/  succinate dehydrogenase | 3,059  3,955  5,253 | 5,149  3,171  2,059 | 15,750  12,541  10,816 |
| GSU1465 | *icd* | isocitrate dehydrogenase | 3,114 | 3,548 | 10,050 |
| GSU1466 | *mdh* | malate dehydrogenase | 4,154 | 2,385 | 9,910 |
| GSU1467  GSU1468  GSU1469  GSU1470 | *korD*  *korA*  *korB*  *korC* | oxoglutarate oxidoreductase | 3,782  4,530  5,302  5,219 | 3,395  3,058  2,254  1,667 | 12,839  13,855  11,952  8,703 |
| GSU2428 | *pyc* | pyruvate carboxylase | 2,608 | 4,118 | 10,741 |
| GSU2445 |  | aconitase | 2,890 | 2,799 | 8,088 |
| GSU2706 | *pta* | phosphate transacetylase | 1,192 | 4,476 | 5,334 |
| GSU2707 | *ackA* | acetate kinase/  propionate kinase | -1,044 | 10,477 | 10,040 |
| GSU3448 |  | acetate kinase  like protein | 2,446 | -1,261 | 1,939 |

*Supplementary Figure 1: FISH analysis of the anode and the planktonic phase of the augmented community in the continuous-mode reactor. Samples were viewed on a Leica DM 5500B, and images were taken with a Leica DFC 360 FX camera and the corresponding Leica LAS AF Lite software. (A) – (D) Images taken from the anode. All cells were stained with DAPI (blue). Arrows indicate* E. coli *cells (red, Cy-3). (E) and (F) Images taken from the planktonic phase.* G. sulfurreducens*_bc_ was stained with Geo_2-probe (green - FITC), E. coli with Ent-probe (red, Cy-3),* Clostridium *sp. I and II and* Paenibacillus *sp. with LGC-probe (yellow, Cy-5). Images of the anodes were taken with Dip-In-lens and 63fold magnification; size bar indicates 50 µm. Images of the planktonic phase were taken with oil immersion lens and 100fold magnification; size bar indicates 25 µm.*
